# Antibiotic treatment reveals the contributions of the gut microbiome to CLN2 disease in the central and enteric nervous system

**DOI:** 10.1038/s41598-026-49850-z

**Published:** 2026-04-25

**Authors:** Ewa A. Ziółkowska, Paris Nix, Bartłomiej Olszowy, Letitia L. Williams, Elizabeth M. Eultgen, Agnieszka Nowacka, Marta Celorrio, Stuart H Friess, Robert O. Heuckeroth, Jonathan D. Cooper

**Affiliations:** 1 Department of Pediatrics, School of Medicine, Washington University in St. Louis, 660 S Euclid Ave, St. Louis, MO 63110 USA; 2 Present Address: Department of Animal Biotechnology and Genetics, Faculty of Animal Breeding and Biology, Bydgoszcz University of Science and Technology, ul. Mazowiecka 28, 85-084 Bydgoszcz, Poland; 3 Department of Medicine, Endocrinology, Edison Family Center for Genome Sciences and Systems Biology, Washington University in St. Louis, School of Medicine, St. Louis, MO 63110 USA; 4 Department of Neurosurgery, Nicolas Copernicus University in Toruń, Collegium Modicum in Bydgoszcz, ul. Curie Skłodowskiej 9, 85-094 Bydgoszcz, Poland; 5 Children’s Hospital of Philadelphia Research Institute, Children’s Hospital of Philadelphia (CHOP), Philadelphia, PA 19104 USA; 6 Perelman School of Medicine, University of Pennsylvania, Philadelphia, PA 19104 USA; 7 Department of Neurology, Washington University in St. Louis, School of Medicine, St. Louis, MO 63110 USA; 8 Department of Genetics, Washington University in St. Louis, School of Medicine, St. Louis, MO 63110 USA

**Keywords:** Neuronal ceroid lipofuscinosis, Gut microbiota, Enteric nervous system, Neuroinflammation, Antibiotic treatment, Brain–gut axis, Diseases, Gastroenterology, Microbiology, Neurology, Neuroscience

## Abstract

The Neuronal Ceroid Lipofuscinoses (NCLs) are fatal inherited lysosomal storage diseases, with pronounced neuron loss in the central nervous system (CNS). Gastrointestinal issues are frequently reported by people with NCLs, although mechanisms underlying these symptoms are poorly understood. We recently demonstrated degeneration occurs within the enteric nervous system (ENS) in several NCLs. Given that the gut microbiome has been shown to be altered a CLN2 mouse model (*Tpp1*^*R207X/R207X*^) and may potentially influence both CNS and ENS pathology, we investigated the long-term impact of modulating the gut microbiome in these mice. This was done by administering a VNAM antibiotic cocktail (vancomycin, neomycin, ampicillin, and metronidazole) for 1-week post-weaning, examining its effects at disease endstage. While VNAM treatment markedly altered the gut microbiome and caused significant loss of enteric neurons in wildtype mice, it did not exacerbate key pathological parameters in either bowel or brain of *Tpp1*^*R207X/R207X*^ mice. These included histomorphometric changes in the small intestine and neurodegeneration of enteric neurons, or CNS neuropathology. However, we did find evidence for moderate protective effects of VNAM upon enteric neurons in the ileum, and upon CNS microglia, but all other pathologies were unaltered in *Tpp1*^*R207X/R207X*^ mice. These findings suggest that intestinal and ENS pathology is primarily driven by TPP1-deficiency rather than changes in the gut microbiome. Indeed, these alterations to the gut microbiome may occur secondary to the impact of CLN2 disease upon the bowel.

**Supplementary Information:**

The online version contains supplementary material available at 10.1038/s41598-026-49850-z.

## Introduction

CLN2 disease (Neuronal ceroid lipofuscinosis type 2, or late infantile Batten disease) is a rare, autosomal recessive neurodegenerative lysosomal storage disorder^1–4^. CLN2 disease is caused by mutations in the *TPP1* gene^5^, leading to deficiency of the lysosomal protease tripeptidyl peptidase 1 (TPP1). This deficiency results in lysosomal dysfunction and the intralysosomal accumulation of storage material^1–3^. Through mechanisms that remain unclear, TPP1 deficiency leads to progressive degeneration of central nervous system (CNS) neurons^6–9^ and a severe neurological disease including seizures, dementia, visual failure and premature death^1–4^. In 2017 the FDA approved intracerebroventricular (ICV) enzyme replacement therapy with cerliponase alfa as a treatment for CLN2 disease^10^, which slows disease progression^11,12^, but does not result in a cure. Incomplete treatment of CLN2 disease may occur partly because CNS-directed ERT does not treat the effects of TPP1 deficiency in the rest of the body. There is increasing anecdotal evidence of gastrointestinal (GI) symptoms in multiple forms of NCL, although little is formally published on this subject^4,13^. Recently we explored the underlying mechanisms and reported progressive degeneration of enteric nervous system (ENS) neurons and bowel dysmotility in a CLN2 disease mouse model (*Tpp1*^*R207X/R207X*^)^14^. These data suggest GI symptoms may at least in part be due to direct effects of CLN2 disease upon the bowel. This may plausibly include changes to the gut microbiome, which have been reported in *Tpp1*^*R207X/R207X*^ mice^15,16^. These alterations in the gut microbiome are not significant in terms of alpha diversity, but did include significant effects upon beta diversity^15,16^. Such microbiome alterations could also potentially influence CNS neurodegeneration in this disorder, so determining its influence, if any, upon disease-associated events in the bowel and brain is important.

Many studies of disease mechanisms in neurodegenerative diseases found a strong link between the gut microbiome and pathological changes in the CNS^17–22^. The microorganisms living in the gut play a vital role in maintaining the intestinal barrier, regulating local and systemic immune responses including effects upon the CNS^18^. When the balance of the microbiota is disrupted, this can lead to dysbiosis, which may trigger the activation of microglia and astroglia, worsening inflammation in the CNS^17,19–22^. Although the influence of the microbiome has been investigated in several neurodegenerative disorders^17,19–22^, the contribution of an altered intestinal microbiome to the pathogenesis of lysosomal diseases such as CLN2 disease remains poorly understood.

The intestinal microbiome has been previously reported to be altered in *Tpp1*^*R207X/R207X*^ mice^15,16^, raising the question whether such microbial changes influence either CNS or enteric components of CLN2 disease. Rather than directly undertaking complex and costly fecal microbiota transfer or co-housing studies, we sought to obtain initial proof-of-concept by first using an antibiotic ablation approach. In order to transiently ablate the gut microbiome, we orally administered a cocktail of vancomycin, neomycin-sulfate, ampicillin, and metronidazole (abbreviated to VNAM)^23–27^ to *Tpp1*^*R207X/R207X*^ mice. We have utilized this antibiotic cocktail previously in studies to investigate the impact of the gut microbiome on neuropathology in models of traumatic brain injury^23–27^. This widely used VNAM cocktail was chosen for its broad-spectrum activity, targeting Gram-positive and Gram-negative aerobic and anaerobic bacteria, inhibiting bacterial protein synthesis, and eliminating certain protozoa^23–27^. Our goal was to assess the extent to which VNAM-induced gut microbial changes may influence disease progression in both the bowel and brain of *Tpp1*^*R207X/R207X*^ mice.

Microbiota profiling confirmed that VNAM administration markedly reduced bacterial diversity and abundance in *Tpp1*^*R207X/R207X*^ mice, indicating a strong effect of this antibiotic cocktail upon gut microbial composition. In summary, VNAM administration did not exacerbate pathological changes in either the CNS or ENS of *Tpp1*^*R207X/R207X*^ mice, or further impact intestinal architecture. However, among these broadly negative data we did find evidence for a small, but significant, protective effect of VNAM upon ENS neuron density that was restricted to the ileum, and upon CD68 positive microglia in the brain of *Tpp1*^*R207X/R207X*^ mice, but there was no influence of VNAM upon other CLN2 brain pathologies. These data suggest that alterations in the gut microbiome are not a primary driver of neurodegenerative processes in CLN2 disease but does not rule out a modulatory influence on certain selected pathologies. In contrast, one-week exposure of 3-week-old WT mice to this VNAM antibiotic cocktail caused long-lasting damage in their ENS and intestinal morphology. Taken together our data suggest the previously documented dysregulation of the microbiome in *Tpp1*^*R207X/R207X*^ mice, is likely a secondary effect of altered gut motility or other impacts of TPP1-deficiency upon the bowel, rather than these microbiota alterations contributing significantly to CLN2 disease pathology. In contrast, our studies did reveal a profound negative impact of the VNAM cocktail on the bowel of healthy WT mice, severely and significantly affecting both ENS neurons and other bowel structures.

## Materials and methods

### Composition of the antibiotic cocktail

The VNAM antibiotic cocktail consisted of 250 mg vancomycin, 500 mg neomycin-sulfate, 500 mg ampicillin, 500 mg metronidazole dissolved in 10 g of grape Kool-Aid (Kraft Heinz, IL, Chicago)^23–27^ and 400 mL of water.

### Experimental design

**Fig. 1:**
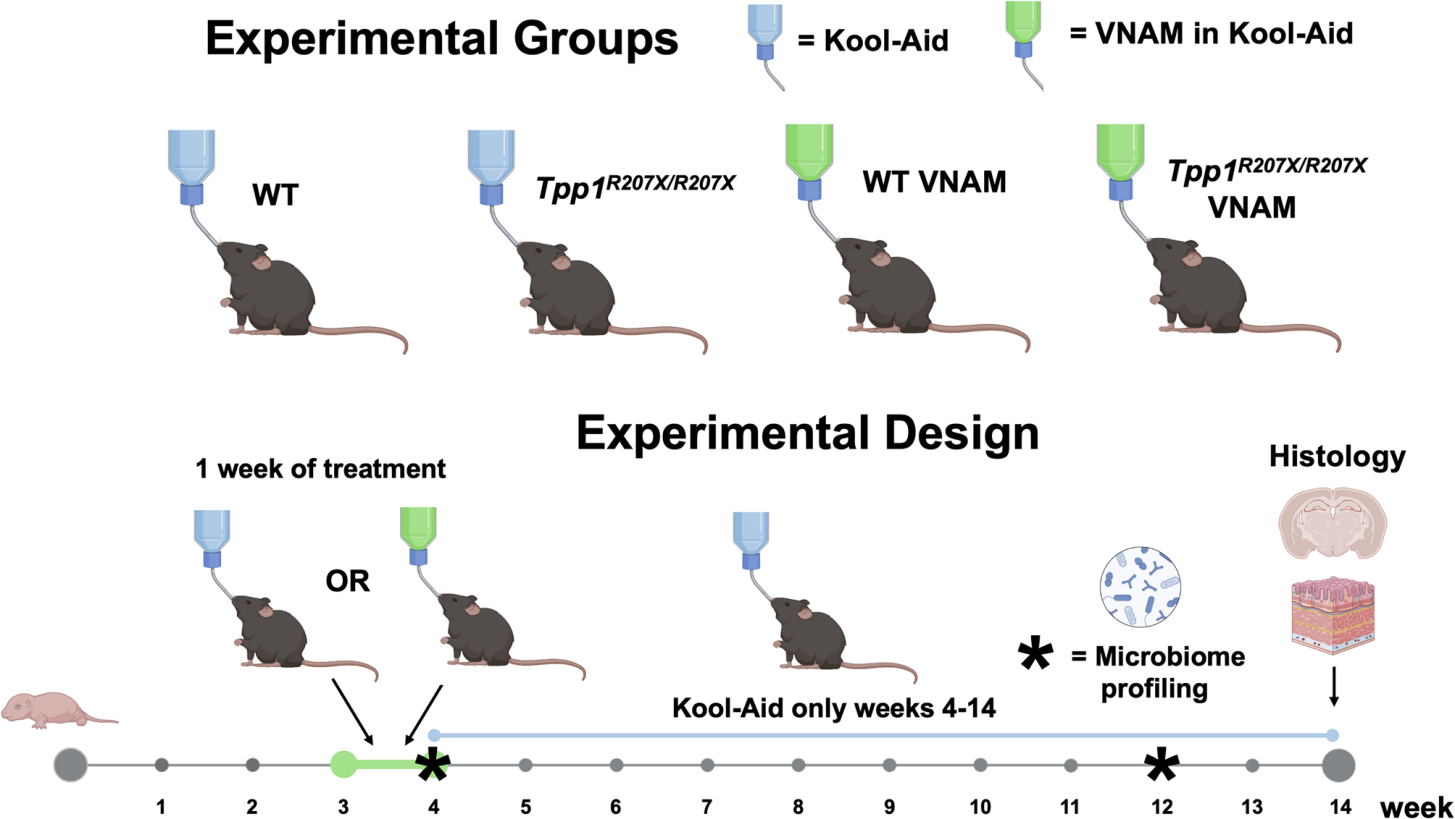
Schematic representation of experimental groups and study design.

As depicted in Fig. 1, after weaning at 3 weeks of age both WT and *Tpp1*^*R207X/R207X*^ mice (*n* = 10) received VNAM antibiotic-supplemented grape Kool-Aid^23–27^ for one week (from week 3 to week 4), or remained on regular grape Kool-Aid (untreated WT and *Tpp1*^*R207X/R207X*^ mice). Each treatment group consisted of two cages of 5 group housed mice, one cage containing 5 male mice and the other containing 5 female mice. This made a total of *n* = 40 mice in the study, with 10 wildtype mice (5 male and 5 female) and 10 *Tpp1*^*R207X/R207X*^ mice (5 male and 5 female) receiving regular grape Kool-Aid, plus 10 wildtype mice (5 male and 5 female) and 10 *Tpp1*^*R207X/R207X*^ mice (5 male and 5 female) receiving VNAM antibiotic-supplemented grape Kool-Aid. After the period of VNAM administration, mice from all treatment groups continued to be group housed in cages of either 5 male or 5 female mice and were provided with regular grape Kool-Aid until the experimental endpoint at 14 weeks of age, corresponding to disease endstage in *Tpp1*^*R207X/R207X*^ mice^28,29^. At this age all mice were sacrificed for bowel^14^ and brain histology^29^, as described below. We did not seek to define effects of TPP1 deficiency upon the gut microbiota, but to confirm whether VNAM administration had altered its composition fecal pellets were collected from each cage of mice in all treatment groups at 4 weeks of age (immediately after VNAM administration ended) or 12 weeks of age (when *Tpp1*^*R207X/R207X*^ mice are severely affected)^28,29^ and pooled fecal samples from each cage were frozen before microbiome analysis (see below).

The top panel illustrates the four experimental groups: wildtype mice (WT), homozygous *Tpp1*^*R207X/R207X*^ mice (a model of CLN2 disease, bearing the most common human disease-causing mutation), and their respective counterparts receiving VNAM antibiotic treatment via drinking water (WT VNAM and *Tpp1*^*R207X/R207X*^ VNAM). As described in the main text, each treatment group consisted of two cages of 5 group housed mice, one containing 5 male mice and the other containing 5 female mice. The bottom panel depicts the experimental timeline: all mice were weaned at 3 weeks of age, at which point the VNAM-treated groups received antibiotic-supplemented grape Kool-Aid for one week (from week 3 to week 4). Afterward, all animals were provided with regular grape Kool-Aid until the experimental endpoint at 14 weeks of age, corresponding to the disease endstage in this CLN2 mouse model, when brain and bowel histology was analyzed. Blue water bottles indicate regular grape Kool-Aid, while green bottles represent grape Kool-Aid supplemented with VNAM. The gut microbiome was profiled in fecal pellets collected at 4 weeks (immediately after VNAM treatment) and at 12 weeks when *Tpp1*^*R207X/R207X*^ mice are severely affected.

This design allowed us to independently assess the impact of VNAM vs. Kool-Aid separately in mice of either sex according to genotype. Our prime readouts of the effect of this transient VNAM administration were well-defined CLN2-disease associated pathologies in the bowel^14^ and brain^29^, that we have characterized previously and shown to be responsive to AAV-mediated gene therapy^14,29^.

### Mice

Both WT and *Tpp1*^*R207X/R207X*^ mice^28^ were maintained separately on a congenic C57Bl/6J background at Washington University School of Medicine. These *Tpp1*^*R207X/R207X*^ mice were originally supplied by Dr. Jill Weimer, Sanford Research Sioux Falls, SD. Mice were provided food and grape Kool-Aid ad libitum under a 12 h light/dark cycle. These studies follow ARRIVE guidelines, were conducted in accordance with NIH guidelines and approved by the Institutional Animal Care and Use Committee (IACUC) at Washington University School of Medicine in St. Louis, MO under protocol 2024 − 0232. The numbers analyzed are detailed in the Figures, but all studies used balanced numbers of males and females.

### 16 S rRNA gene sequencing and microbiome analysis

To confirm whether VNAM administration had altered the gut microbiome fecal pellets were collected and pooled from each cage of mice and sent to Molecular Research, LP (MR DNA, Shallowater, TX, USA) for DNA extraction, sequencing, and microbiome analysis^24–27^. 16 S rRNA gene sequencing was performed using the Illumina MiSeq platform with a modified version of the bTEFAP^®^ method, which amplifies the V4 region of the 16 S rRNA gene. PCR primers 515 F (GTGYCAGCMGCCGCGGTAA) and 806R (GGACTACNVGGGTWTCTAAT) were used for amplification in a 30-cycle reaction (5 cycles used on PCR products), employing the AllTaq Master Mix Kit (Qiagen, USA). PCR conditions were as follows: 95 °C for 5 min, followed by 30 cycles of 95 °C for 30 s, 53 °C for 40 s, and 72 °C for 1 min, with a final elongation step at 72 °C for 10 min. The amplified PCR products were checked via gel electrophoresis for amplification success and relative band intensity. The samples were then multiplexed using unique dual indices, pooled in equal proportions based on molecular weight and DNA concentration, and purified using Ampure XP beads (Beckman Coulter, Brea, CA). The pooled and purified PCR products were used to prepare an Illumina DNA library. Sequencing was performed by MR DNA using the Illumina MiSeq system following their standard protocol. Sequence data were processed through the MR DNA analysis pipeline, which involved quality control steps including the removal of primer sequences, exclusion of sequences shorter than 150 bp, and removal of sequences with ambiguous base calls. The sequences were then quality-filtered, dereplicated, and denoised. Chimeric sequences were removed, resulting in denoised sequences known as zOTUs (zero-radius operational taxonomic units), which provide a higher resolution of microbial diversity by distinguishing sequences with even a single base pair difference. The final zOTUs were taxonomically classified using BLASTn against a curated NCBI-derived database, and the sequences were quantified in both absolute counts and relative percentages to determine the microbiota composition. Using zOTUs over traditional OTUs allows for greater specificity in identifying microbial species or strains with high sequence similarity. Alpha diversity metrics, including Observed Features and the Shannon Diversity Index, were calculated to assess the richness and evenness of microbial communities within each sample. Beta diversity was analyzed using Unweighted and Weighted UniFrac, Bray-Curtis, and Jaccard distances to assess dissimilarity between samples. PCoA plots and other visualizations were generated to represent the diversity metrics. However, we did not seek to assess any statistical significance to any changes in gut microbiome composition as this study was neither designed nor sufficiently powered to make such comparisons valid. All data were processed and visualized using the Qiime2 pipeline (https://qiime2.org/), and interactive visualizations of alpha and beta diversity were made available using the Qiime2 View tool (https://view.qiime2.org/)^30^. The microbial diversity data were processed by MR DNA, and the results, including alpha and beta diversity metrics as well as taxonomic classification, were imported into R for visualization using the microViz package. Alpha diversity metrics, including the Shannon Diversity Index and Observed Features, were visualized using boxplots to compare microbial richness and evenness across experimental groups. Beta diversity was assessed by visualizing principal coordinate analysis (PCoA) plots based on distance matrices calculated using Unweighted UniFrac, Weighted UniFrac, Bray-Curtis, and Jaccard indices. These PCoA plots were used to examine the clustering and separation of samples from different experimental conditions. Additionally, the relative abundance of microbial taxa was visualized using taxa-bar-plots and heatmaps, displaying the distribution of taxa at various taxonomic levels (e.g., phylum, genus) across the samples. These visualizations facilitated the identification of compositional differences between groups and were generated using microViz and refined using ggplot2 to enhance the graphical presentation.

### Wholemount bowel histology in mice

Mice anesthetized (2% isoflurane) were transcardially perfused with PBS and decapitated. The first and last 8 cm of small intestine and entire colon were cut into 2 cm lengths in cold 50 mM Tris buffered saline (TBS, pH = 7.6), opened along mesenteric border, pinned to Sylgard™ 184 Silicone Elastomer (Dow Corning) using stainless steel insect pins, and fixed in fresh 4% paraformaldehyde (35 min colon, 45 min ileum, 1 h duodenum)^14,31–33^. Fixed bowel tissue was cut into 1 cm lengths and stored in TBS/0.1% sodium azide (4 °C) before staining for neuronal or glial markers^14,31–33^. Muscle layers were separated from mucosa and submucosa and blocked (1 h) in TBS containing 4% Triton X-100 (TBST), 15% normal goat serum (NGS) (Jackson Immuno-Research Laboratories), then incubated in primary antibody (Supplemental Table 1) at 4 °C overnight, washed (3x, TBS), and incubated in secondary antibodies (Supplemental Table 1) in 10% goat serum, 4% TBST for 2 hours^14,31–33^. Bowel wholemounts were mounted on Colorfrost plus (Thermo Fisher Scientific) slides, dried briefly before incubation in 1x solution TrueBlack lipofuscin autofluorescence quencher (Biotium) in 70% ethanol (5 min) and rinsing (1xTBS). Slides were coverslipped in Fluoromount-G mounting medium containing DAPI (Southern Biotech).

### Quantitative analysis of immunostained mouse bowel

For each sample, 10 systematically sampled, regularly spaced 20× fields (each 0.42250 mm^2^) per sample were analyzed^14,31–33^. Multi-channel images were collected on a Zeiss AxioImager Z1 microscope using StereoInvestigator (MBF Bioscience) software and exported to ImageJ (NIH). Myenteric neurons (HuC/D+ cells) and enteric glia (S100B+ cells) were manually counted in each field and averaged to determine cells/mm^2,14,31–33^. All analyses were made blind to genotype or treatment.

### Mouse brain processing and immunostaining

Brains removed from the same mice euthanized for bowel preparations were bisected. One hemisphere was drop fixed for 48 h in fresh 4% paraformaldehyde, then cryoprotected (30% sucrose in 50 mM TBS, pH = 7.6)^14,29^. Forty µm coronal forebrain sections were cut using a Microm HM430 freezing microtome (Microm International) equipped with a Physitemp BFS-40MPA freezing stage (Physitemp, Clifton, NJ). Sections were collected into 96-well plates containing cryoprotectant solution, as described^14,29^.

A one-in-six series of coronal forebrain sections from each mouse was stained on slides using a modified immunofluorescence protocol employing TrueBlack and stained with GFAP, CD68 and SCMAS antibodies^14,29^. To quantify storage accumulation (SCMAS+) and glial activation (GFAP+ astrocytes, CD68 + microglia), thresholding image analysis was performed as described using slide-scanned images at 10x magnification (Zeiss AxioScan Z1)^14,29^. Contours of appropriate anatomical regions were drawn and images analyzed using *Image-Pro Premier* (Media Cybernetics) and thresholds selected for foreground immunoreactivity above background.

### Bowel histology and analyses

Immediately after decapitation, jejunum was harvested and fixed overnight at 4 °C in fresh 4% paraformaldehyde. Fixed pieces of jejunum were embedded in paraffin blocks and cross sectioned at 10 μm thickness using a sledge microtome. Sections were deparaffinized and stained with hematoxylin and eosin^34,35^. Then, measurements were made of the height and width of the intestinal villi, the depth of the crypts, and the thickness of the muscular layer in at least 5 sections per individual, with 50 measurements made in each section.^34,35^.

### Statistical analyses

Statistical analyses of bowel and CNS histology were performed using GraphPad Prism version 9.1.0 for MacOS (GraphPad Software, San Diego, CA). Unpaired t-tests were used for comparison between two groups based on data distributions. A two-way ANOVA with a post-hoc Bonferroni correction was used for comparison between groups using a 2 × 2 design to assess genotype × treatment effects. A *p* value of ≤ 0.05 was considered significant.

## Results

### Gut microbiome composition and diversity are disrupted by VNAM treatment in both WT and *Tpp1*^*R207X/R207X*^ mice

**Fig. 2:**
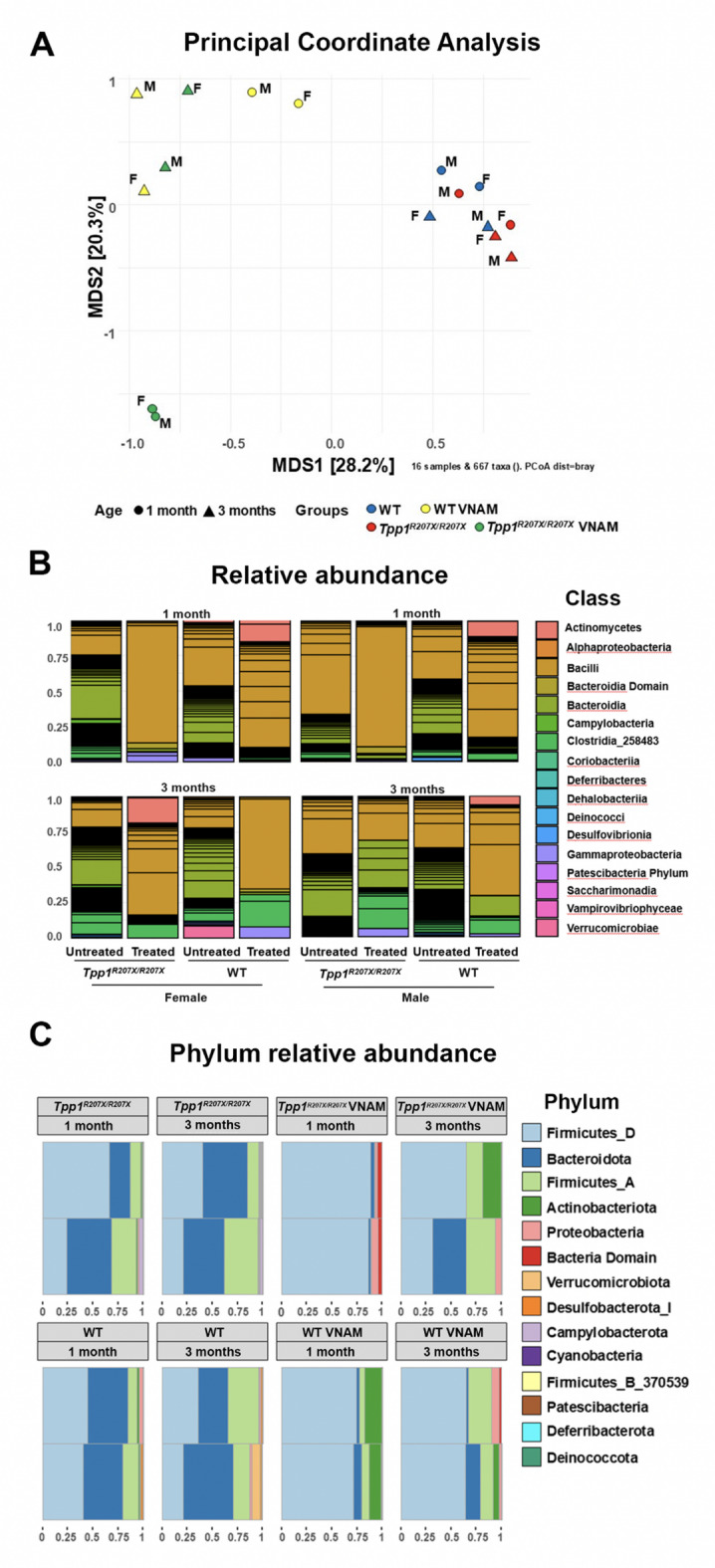
Microbial community composition of *Tpp1*^*R207X/R207X*^ mice and the impact of VNAM treatment. (**A**) Principal coordinate analysis (PCoA) of Bray–Curtis dissimilarity among fecal samples shows clustering by genotype and treatment. Each point represents data obtained from a pooled sample of fecal pellets taken from a single cage of *n* = 5 mice (either male or female), colored by group (WT, *Tpp1*^*R207X/R207X*^, WT+VNAM, and *Tpp1*^*R207X/R207X*^+VNAM) and shaped by age (1 or 3 months, corresponding to just after VNAM administration and 2 months later). (**B**) Stacked bar plots showing the relative abundance of bacterial classes in individual fecal samples, grouped by sex and age. Each bar represents a sample, and segments are colored by bacterial class. (**C**) Relative abundance of bacterial phyla across fecal samples from each experimental group. Bars represent individual samples (replicate cages), with the x-axis showing relative abundance (0–1) and the y-axis indicating replicate cages. The bars are colored according to taxonomic phylum and facet-wrapped by group (WT, *Tpp1*^*R207X/R207X*^, WT+VNAM, and *Tpp1*^*R207X/R207X*^+VNAM) and age.

Dysregulation of the gut microbiome has previously been documented in *Tpp1*^*R207X/R207X*^ mice^15,16^. In this study we performed gut microbiome profiling solely to confirm that transient administration of VNAM had impacted the intestinal microbiome, and whether these effects were long lasting. To do this we performed 16 S rRNA gene sequencing of pooled fecal samples collected from each cage of either male or female WT and *Tpp1*^*R207X/R207X*^ mice at 4 and 12 weeks of age, with or without exposure to the VNAM antibiotic cocktail (vancomycin, neomycin, ampicillin, and metronidazole)^23–27^. Principal coordinates analysis (PCoA) based on Bray-Curtis dissimilarities revealed distinct clustering patterns according to genotype and antibiotic treatment (Fig. 2A). VNAM-treated animals formed discrete clusters, reflecting a strong shift in microbial composition that appeared to supersede genotype- and sex-associated variation (Fig. 2B). These patterns were generally consistent across sexes and developmental timepoints, but phylum-level analysis further suggested decreased diversity in *Tpp1*^*R207X/R207X*^ mice (Fig. 2C),

### Intestinal structural alterations in *Tpp1*^*R207X/R207X*^ mice are not altered by VNAM treatment and are exacerbated in wildtype mice

The small intestine performs many of its functions through the epithelial cells that line villi and the intestinal crypts of Lieberkühn^36–38^. Intestinal villi, with their large surface area and length, are the primary sites of nutrient absorption^38^. In contrast, the crypts house stem cells responsible for the continuous renewal of the bowel epithelium through cell proliferation and differentiation^36,38^. The depth of the crypts correlates with the intensity of these regenerative processes. Any disturbances in these structures, whether in the villi or crypts, can impair intestinal function and disrupt the overall homeostasis of the organism^36–38^. To determine whether any morphological parameters of intestinal villi and crypts were altered by TPP1 deficiency or VNAM administration we performed quantitative analyses of H&E-stained sections at disease endstage (14 weeks). Quantified parameters included villus height, villus width, villus surface area, crypt depth, villus height to crypt depth ratio, along with measurements of circular muscle thickness, longitudinal muscle thickness, and total tunica muscularis thickness.

These analyses revealed that untreated *Tpp1*^*R207X/R207X*^ mice had significantly shorter villus height, reduced villus surface area, and reduced villus height to crypt depth ratio compared to WT mice. The thickness of the circular layer of bowel smooth muscle was also reduced, but not significantly, whereas the thickness of the longitudinal muscle layer and total bowel smooth muscle layer was significantly reduced in untreated *Tpp1*^*R207X/R207X*^ mice versus WT mice. Administration of the antibiotic VNAM to *Tpp1*^*R207X/R207X*^ mice did not cause any additional significant changes in any structural parameter of the intestinal villi and crypts of untreated *Tpp1*^*R207X/R207X*^ mice, and VNAM did not alter bowel smooth muscle thickness in *Tpp1*^*R207X/R207X*^ or WT mice.

**Fig. 3:**
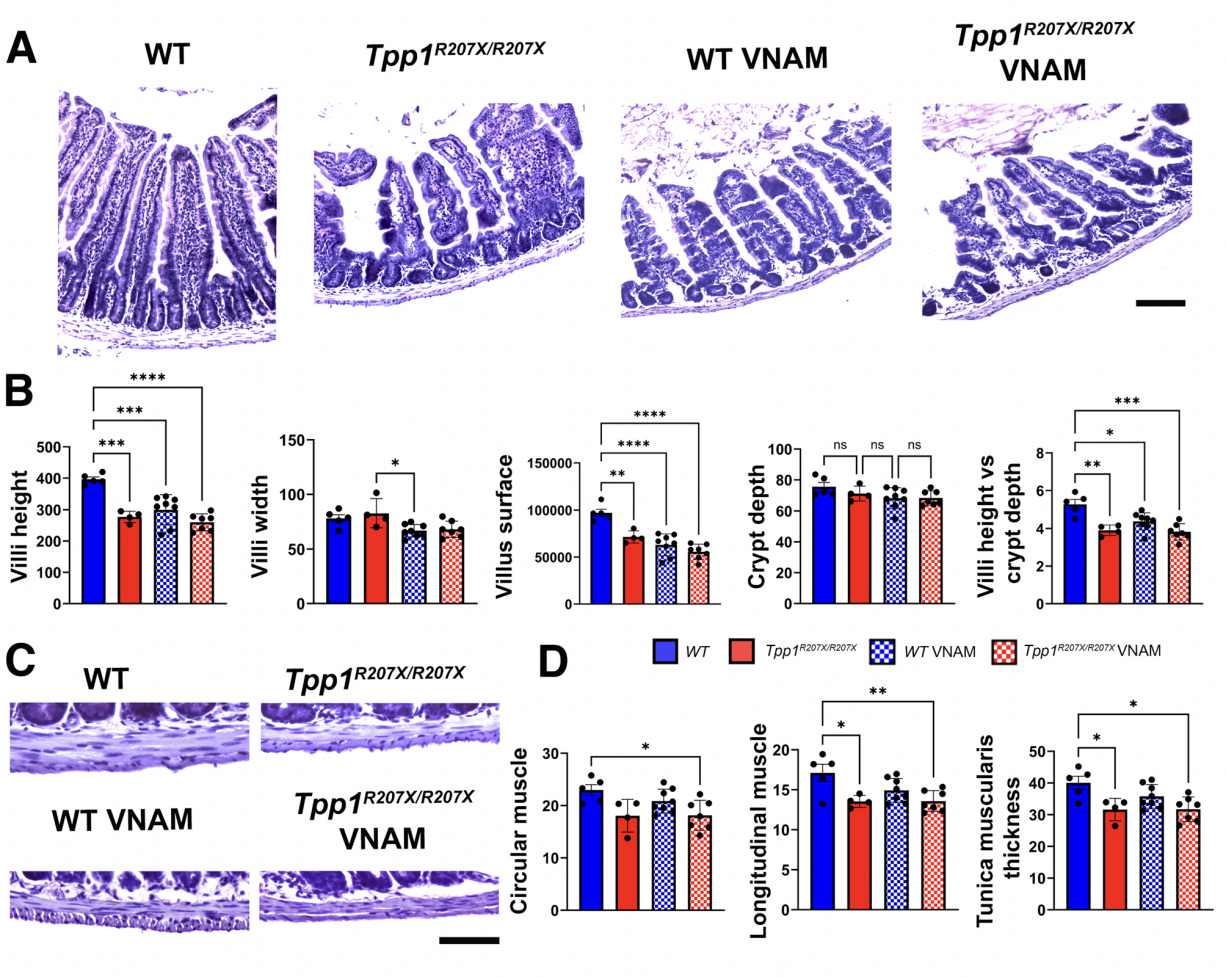
Histopathological changes in the small intestine of *Tpp1*^*R207X/R207X*^ mice and the impact of VNAM treatment. (**A**) Representative H&E-stained sections of the jejunum of disease endstage *Tpp1*^*R207X/R207X*^ mice at 14 weeks reveal the impact of TPP1 deficiency on the morphology of both villi and crypts. Scale bar = 200 μm. (**B**) Histograms depicting quantification of villus height and width, villi surface area, crypt depth and ratio of villi height to crypt depth across treatment groups. Compared to WT, TPP1 deficiency reduced villus height, villus surface area and villi height to crypt depth ratio but none of these phenotypes were further exacerbated by VNAM. In contrast, VNAM administration to WT mice significantly compromised these morphological parameters. (**C**) Representative H&E-stained sections of the small intestine reveal the moderate impact of TPP1 deficiency on bowel smooth muscle. Scale bar = 50 μm. (**D**) Histograms depicting the quantification of the thickness of the circular and longitudinal smooth muscle layers and a combined measure of overall tunica muscularis thickness across treatment groups. Statistical analysis was performed using two-way ANOVA followed by Bonferroni post hoc test. *p ≤ 0.05, **p ≤ 0.01, ***p ≤ 0.001, ****p ≤ 0.0001. Data are presented as mean ± SEM.

In marked contrast, VNAM administration to WT mice caused significant changes in many quantified parameters of intestinal structure, yielding anatomy that closely resembled CLN2 disease mice with or without VNAM treatment (Fig. 3). Comparing VNAM treated to untreated WT mice, these effects included significantly reduced villi height, reduced villus surface area, and reduced villi height to crypt depth ratio. Although VNAM treatment reduced the thickness of both circular and longitudinal smooth muscle layers and total bowel smooth muscle thickness, none of these effects were significant.

Taken together these data suggest that many parameters of intestinal morphology are compromised by TPP1 deficiency, but none of these are exacerbated further by VNAM administration. In contrast, this transient administration of VNAM severely impairs intestinal morphology in WT mice, causing long-lasting deleterious effects.

### CLN2-related ENS damage is not exacerbated by microbiota disruption with VNAM

**Fig. 4:**
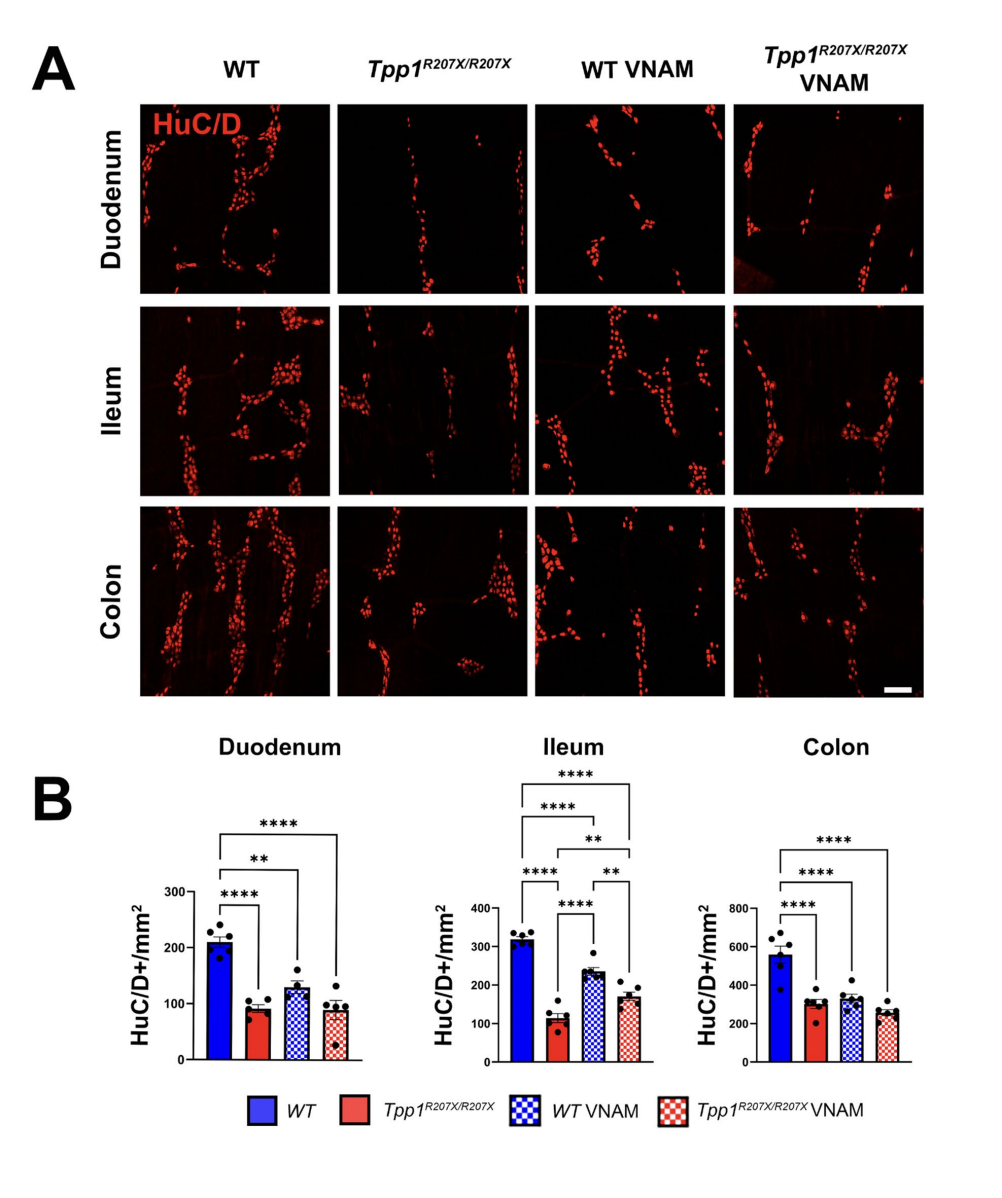
Enteric neuron loss in *Tpp1*^*R207X/R207X*^ mice is not exacerbated by VNAM treatment (**A**) Immunostaining for the enteric neuron marker HuC/D (red) reveals a significant reduction in the density of enteric neurons within the myenteric plexus of the duodenum, ileum, and colon of untreated *Tpp1*^*R207X/R207X*^ mice, and VNAM treated WT mice (WT+VNAM) compared to WT controls at 14 weeks of age. However, VNAM treatment of *Tpp1*^*R207X/R207X*^ mice (Tpp1^R207X/R207X^+VNAM mice), does not cause any additional enteric neuron loss. (**B**) Quantification of HuC/D+ neuron density demonstrates similar significant neuronal loss across all intestinal regions in untreated *Tpp1*^*R207X/R207X*^ mice, VNAM treated *Tpp1*^*R207X/R207X*^ mice (Tpp1^R207X/R207X^+VNAM) and VNAM treated WT mice (WT+VNAM). Statistical analysis was performed using two-way ANOVA followed by Bonferroni post hoc test. *p ≤ 0.05, **p ≤ 0.01, ***p ≤ 0.001, ****p ≤ 0.0001. Data are presented as mean ± SEM. (*n* = 6 mice per treatment group). Scale bar = 200 μm.

The ENS is an autonomous network of neurons and glia that regulates many aspects of bowel function including gastrointestinal motility, epithelial biology, and immune cell function^39–43^. ENS neurons play a pivotal role in maintaining intestinal homeostasis and coordinating peristalsis, and their degeneration can precipitate bowel dysmotility^39–43^, as we recently reported in multiple forms of NCL^14,35^, including CLN2 disease. To determine whether disruption of the gut microbiome influenced enteric neuron survival in the myenteric plexus we immunostained bowel wholemounts from the duodenum, jejunum, and colon of untreated and VNAM treated *Tpp1*^*R207X/R207X*^ and WT mice at 14 weeks of age for the neuronal marker HuC/D^14,35^ (Fig. 4A). Quantitative analysis confirmed the significant reduction in density of HuC/D positive myenteric plexus neurons in all bowel regions of untreated *Tpp1*^*R207X/R207X*^ mice compared to WT mice, substantiating our previous observations^14^ (Fig. 4B; Table 1).

**Table 1:** Density of HuC/D+ enteric neurons in untreated and VNAM treated *Tpp1*^*R207X/R207X*^ and wildtype (WT) mice at disease endstage (mean ± SEM), percent loss, and *p* values.

HuC/D	WT neurons/mm^2^	*Tpp1*^*R207X/R207X*^ neurons/mm^2^	Percent (%) loss	*p* value (WT vs. *Tpp1*^*R207X/R207X*^)
Duodenum	210.13 ± 9.35	91.70 ± 6.83	56.36%	< 0.0001
Ileum	318.87 ± 6.82	114.12 ± 11.61	64.21%	< 0.0001
Colon	558.90 ± 44.29	301.97 ± 23.47	45.97%	< 0.0001

HuC/D	WT neurons/mm^2^	WT VNAM neurons/mm^2^	Percent (%) loss	*p* value (WT vs. WT VNAM)
Duodenum	210.13 ± 9.35	130.24 ± 11.38	38.02%	0.0014
Ileum	318.87 ± 6.82	235.35 ± 10.12	26.19%	< 0.0001
Colon	558.90 ± 44.29	330.61 ± 22.46	40.85%	< 0.0001

HuC/D	WT neurons/mm^2^	*Tpp1*^*R207X/R207X*^ VNAM neurons/mm^2^	Percent (%) loss	*p* value (WT vs. *Tpp1*^*R207X/R207X*^ VNAM)
Duodenum	210.13 ± 9.35	89.94 ± 17.21	57.20%	0.0001
Ileum	318.87 ± 6.82	170.57 ± 10.89	46.51%	< 0.0001
Colon	558.90 ± 44.29	256.33 ± 15.83	54.14%	< 0.0001

HuC/D	*Tpp1* ^*R207X/R207X*^ neurons/mm^2^	WT VNAM neurons/mm^2^	Percent (%) loss	*p* value (*Tpp1*^*R207X/R207X*^ vs. WT VNAM)
Duodenum	91.70 ± 6.83	130.24 ± 11.38	− 42.02%	0.2633
Ileum	114.12 ± 11.61	235.35 ± 10.12	− 106.23%	< 0.0001
Colon	301.97 ± 23.47	330.61 ± 22.46	− 9.49%	> 0.9999

HuC/D	*Tpp1* ^*R207X/R207X*^ neurons/mm^2^	*Tpp1*^*R207X/R207X*^ VNAM neurons/mm^2^	Percent (%) loss	*p* value (*Tpp1*^*R207X/R207X*^ vs. *Tpp1*^*R207X/R207X*^ VNAM)
Duodenum	91.70 ± 6.83	89.94 ± 17.21	1.92%	> 0.9999
Ileum	114.12 ± 11.61	170.57 ± 10.89	− 49.47%	0.0044
Colon	301.97 ± 23.47	256.33 ± 15.83	− 15.11%	> 0.9999

**Fig. 5:**
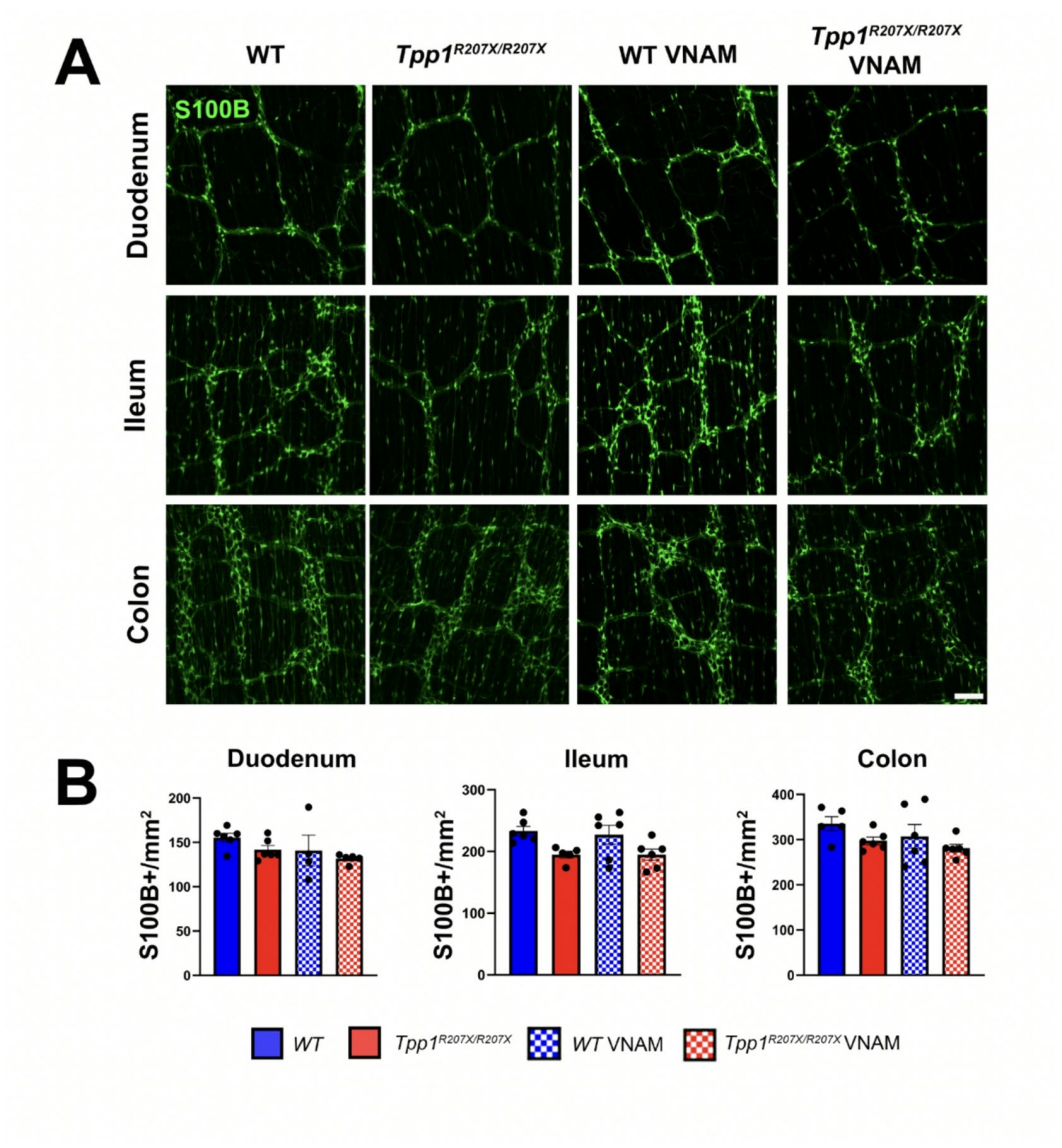
Enteric glial cell density is preserved in the gastrointestinal tract of VNAM-treated and untreated *Tpp1*^*R207X/R207X*^ mice. (**A**) Immunostaining for the enteric glial cell marker S100B (green) shows only moderate differences in the density or distribution of enteric glia in the myenteric plexus of the duodenum, ileum, and colon among untreated WT, untreated *Tpp1*^*R207X/R207X*^, VNAM treated WT (WT+VNAM) and VNAM treated *Tpp1*^*R207X/R207X*^ mice (*Tpp1*^*R207X/R207X*^+VNAM) mice at 14 weeks of age. (**B**) Quantitative analysis of S100B+ cell density per mm² confirms that enteric glial cell numbers are not significantly changed across all genotypes and treatment conditions. Statistical analysis was performed using two-way ANOVA followed by Bonferroni post hoc test. Data are presented as mean ± SEM. (*n* = 6 mice per treatment group). Scale bar = 200 μm.

However, exposure to VNAM did not further worsen the CLN2 disease associated reduced density of HuC/D positive myenteric plexus neurons in any bowel region of VNAM treated *Tpp1*^*R207X/R207X*^ mice (Fig. 4B). Indeed, VNAM treatment had a small, but significant, protective effect upon myenteric plexus neuron density in the ileum of *Tpp1*^*R207X/R207X*^ mice compared to untreated *Tpp1*^*R207X/R207X*^ mice, but this effect was not evident in either the duodenum or colon of these mice. In marked contrast, 1 week of VNAM treatment at weaning resulted in a profound and long-lasting negative impact upon WT mice, significantly reducing enteric neuron density in all bowel regions at 14 weeks of age compared to untreated WT mice of the same age. Indeed, this impact of VNAM treatment was of comparable magnitude to the effect of TPP1 deficiency alone.

We also assessed the impact of VNAM administration on S100B +ve glial cells within muscularis, which play a key role in maintaining neuronal homeostasis and the intestinal barrier^44–48^. These analyses revealed that no significant differences in the density of S100B+ve enteric glia were found between any of the treatment groups (Fig. 5A, B; Table 2), indicating the relative stability of enteric glia, regardless of the presence of TPP1 deficiency or VNAM administration.

**Table 2:** Density of S100B+ enteric glia in untreated and VNAM treated *Tpp1*^*R207X/R207X*^ and wildtype (WT) mice at disease endstage (mean ± SEM), percent loss, and *p* values.

HuC/D	WT VNAM neurons/mm^2^	*Tpp1*^*R207X/R207X*^ VNAM neurons/mm^2^	Percent (%) loss	*p* value (WT VNAM vs. *Tpp1*^*R207X/R207X*^ VNAM)
Duodenum	130.24 ± 11.38	89.94 ± 17.21	30.94%	0.2167
Ileum	235.35 ± 10.12	170.57 ± 10.89	27.52%	0.0011
Colon	330.61 ± 22.46	256.33 ± 15.83	22.47%	0.486

S100B	WT S100B+ Ganglionic glia/ mm^2^	*Tpp1*^*R207X/R207X*^ S100B+ Ganglionic glia/ mm^2^	Percent (%) loss	*p* value (WT vs. T*Tpp1*^*R207X/R207X*^)
Duodenum	155.45 ± 4.75	141.77 ± 4.84	8.80%	> 0.9999
Ileum	233.13 ± 7.56	194.80 ± 5.67	16.44%	0.1255
Colon	335.30 ± 14.32	297.37 ± 8.40	11.31%	0.7837

S100B	WT S100B+ Ganglionic glia/ mm^2^	WT VNAM S100B+ Ganglionic glia/ mm^2^	Percent (%) loss	*p* value (WT vs. WT VNAM )
Duodenum	155.45 ± 4.75	140.83 ± 17.45	9.41%	> 0.9999
Ileum	233.13 ± 7.56	227.26 ± 15.49	2.52%	> 0.9999
Colon	335.30 ± 14.32	307.22 ± 26.20	8.37%	> 0.9999

S100B	WT S100B+ Ganglionic glia/ mm^2^	*Tpp1*^*R207X/R207X*^ VNAM S100B+ Ganglionic glia/ mm^2^	Percent (%) loss	*p* value (WT vs. *Tpp1*^*R207X/R207X*^ VNAM)
Duodenum	155.45 ± 4.75	131.64 ± 2.29	15.31%	0.2229
Ileum	233.13 ± 7.56	194.87 ± 9.05	16.41%	0.0976
Colon	335.30 ± 14.32	281.07 ± 8.63	16.18%	0.215

S100B	*Tpp1*^*R207X/R207X*^ S100B+ Ganglionic glia/ mm^2^	WT VNAM S100B+ Ganglionic glia/ mm^2^	Percent (%) loss	*p* value (*Tpp1*^*R207X/R207X*^ vs. WT VNAM)
Duodenum	141.77 ± 4.84	140.83 ± 17.45	0.66%	> 0.9999
Ileum	194.80 ± 5.67	227.26 ± 15.49	− 16.66%	0.2775
Colon	297.37 ± 8.40	307.22 ± 26.20	− 3.31%	> 0.9999

S100B	*Tpp1*^*R207X/R207X*^ S100B+ Ganglionic glia/ mm^2^	*Tpp1*^*R207X/R207X*^ VNAM S100B+ Ganglionic glia/ mm^2^	Percent (%) loss	*p* value (*Tpp1*^*R207X/R207X*^ vs. *Tpp1*^*R207X/R207X*^ VNAM)
Duodenum	141.77 ± 4.84	131.64 ± 2.29	7.14%	> 0.9999
Ileum	194.80 ± 5.67	194.87 ± 9.05	− 0.04%	> 0.9999
Colon	297.37 ± 8.40	281.07 ± 8.63	5.48%	> 0.9999

S100B	WT VNAM S100B+ Ganglionic glia/ mm^2^	*Tpp1*^*R207X/R207X*^ VNAM S100B+ Ganglionic glia/ mm^2^	Percent (%) loss	*p* value(WT VNAM vs. *Tpp1*^*R207X/R207X*^ VNAM)
Duodenum	140.83 ± 17.45	131.64 ± 2.29	6.52%	> 0.9999
Ileum	227.26 ± 15.49	194.87 ± 9.05	14.25%	0.2274
Colon	307.22 ± 26.20	281.07 ± 8.63	8.51%	> 0.9999

Taken together these data from bowel wholemounts reveal that while neurodegeneration in the ENS is clearly associated with TPP1 deficiency, VNAM treatment does not cause further loss of enteric neurons and may even have a small protective effect upon the neurodegenerative effects of TPP1 deficiency in the ileum, but does not affect the density of S100B+ enteric glial cells (Fig. 5). However, even one week of VNAM treatment post weaning has very different effects in WT mice, severely reducing enteric neuron density with effects that persist for many months.

### Effects of VNAM administration upon the brain

**Fig. 6:**
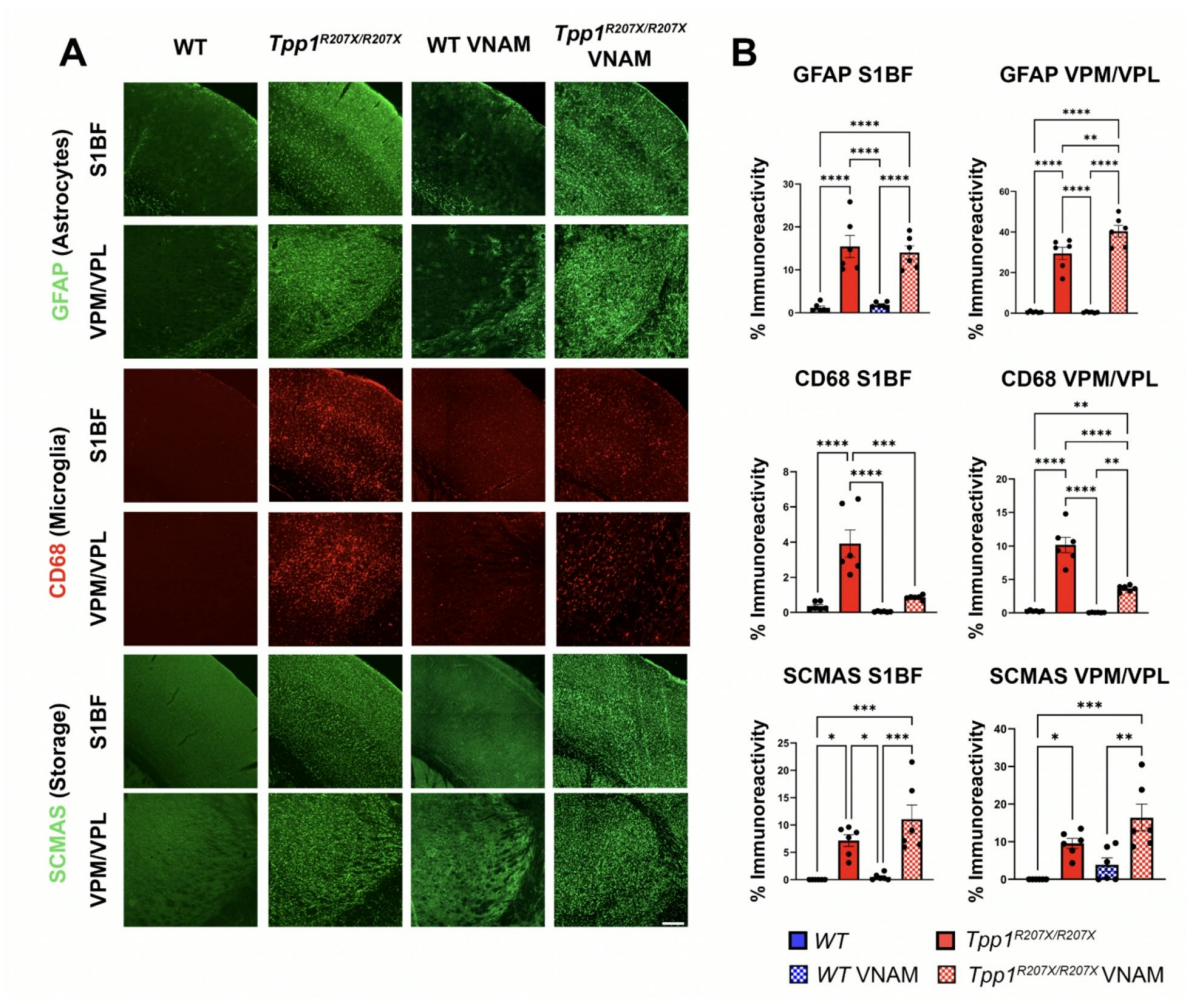
The impact of VNAM upon CNS pathology in *Tpp1*^*R207X/R207X*^ mice. (**A**) Representative images and quantification of immunoreactivity for astrocytes (GFAP, green), microglia (CD68, red), and storage material (subunit C of mitochondrial ATP synthase, SCMAS, green) in the primary somatosensory barrel field (S1BF) and the ventral posteromedial/lateral thalamic nuclei (VPM/VPL) of untreated WT and *Tpp1*^*R207X/R207X*^ mice, and VNAM treated WT (WT+VNAM), and *Tpp1*^*R207X/R207X*^ mice (*Tpp1*^*R207X/R207X*^+VNAM) at 14 weeks of age. (**B**) Quantitative analyses (mean ± SEM) show statistically significant increases in GFAP, CD68, and SCMAS immunoreactivity in untreated *Tpp1*^*R207X/R207X*^ mice relative to WT controls (as published previously^29^), and a significant reduction in CD68 + microglia *Tpp1*^*R207X/R207X*^+VNAM mice compared to untreated *Tpp1*^*R207X/R207X*^ mice, and a small but significant increase in GFAP in the thalamus of *Tpp1*^*R207X/R207X*^+VNAM mice compared to untreated *Tpp1*^*R207X/R207X*^ mice. Statistical significance was assessed using two-way ANOVA with Bonferroni post hoc correction: * *p* < 0.05, **p < 0.01, ***p < 0.001, ****p < 0.0001. (*n* = 6 animals per group). Scale Bar = 200 μm.

The CNS neuropathology of CLN2 disease is characterized by the intralysosomal accumulation of storage material and pronounced microglial activation and astrocytosis^6–9,29^ phenotypes that are often used to assess the efficacy of CNS-directed therapeutic strategies^29^. Given the potential impact of altering the gut.

microbiome upon CNS pathology we assessed whether administration of the VNAM antibiotic cocktail had any effects upon these well-defined CNS phenotypes. This analysis focused on brain regions that we have previously characterized as being most affected by TPP1 deficiency^29^, including the somatosensory relay pathways through the thalamus (ventral posterior nuclei, VPM/VPL) to primary barrel field sensorycortex (S1BF). Our analyses revealed the anticipated significant accumulation of SCMAS positive storage material, GFAP positive astrocytes and CD68 positive microglia in both VPM/VPL and S1BF in untreated *Tpp1*^*R207X/R207X*^ mice compared to WT mice (Fig. 6). VNAM administration had only a moderate additional impact upon storage material accumulation or astrocytosis (Fig. 6A), resulting in SCMAS levels that were statistically indistinguishable from untreated *Tpp1*^*R207X/R207X*^ mice (Fig. 6B). The levels of GFAP in S1BF of *Tpp1*^*R207X/R207X*^ mice were not statistically altered by VNAM treatment, but astrocytosis in the thalamus was significantly elevated in VNAM treated *Tpp1*^*R207X/R207X*^ mice (Fig. 6B). However, VNAM treatment markedly reduced the abundance of CD68 positive microglia in *Tpp1*^*R207X/R207X*^ mice, although CD68 + cells remained elevated compared to WT mice (Fig. 6B). In contrast, VNAM treatment did not produce any overt effect upon astrocytosis or microglial activation in WT mice (Fig. 6A), but a small and not significant elevation in storage material in the thalamus (Fig. 6B).

These findings indicate that while VNAM treatment does not affect lysosomal storage accumulation in CLN2 mice, it selectively modulates the neuroinflammatory landscape—particularly by partially reducing CD68 + microglial activation in the brain. Furthermore, unlike the bowel, VNAM administration produced no negative impact upon measured parameters in the brain of WT mice.

## Discussion

Based on findings in many other disorders, the gut microbiome (community of microorganisms inhabiting the intestines) may influence neurodegeneration within both brain and bowel^17–22^. The gut microbiome is markedly altered in *Tpp1*^*R207X/R207X*^ mice^15,16^ so this study was designed to investigate the potential influence of modulating gut microbes upon bowel and brain pathology in CLN2 disease. Microbes were depleted via a short period of VNAM administration^23–27^ which profoundly impacted the ENS and bowel morphology in wildtype mice but did not worsen the effects of TPP1 deficiency upon enteric neuron or glial cell density, or epithelial anatomy in *Tpp1*^*R207X/R207X*^ mice. Indeed, VNAM had a moderate protective effect on enteric neuron density that was confined to the ileum but was not present in other bowel regions. In the CNS, VNAM administration again had no negative impact upon disease-associated brain pathology in *Tpp1*^*R207X/R207X*^ mice, but did partly reduce CD68 + microglia in the brain and did not impact other disease-associated CNS pathologies. Taken together these data suggest that the dysregulated microbiome present in *Tpp1*^*R207X/R207X*^ mice is not a primary driver of bowel disease but may instead be a consequence of the recently identified ENS defects in CLN2 disease^14^.

The role of the gut-brain axis (GBA) in CNS neurodegeneration has come under renewed scrutiny in recent years with evidence building in several disorders^17–22^. Perhaps best recognized is the seeding of synuclein pathology within enteric neurons, with subsequent spread of synuclein aggregates to the brain via the vagus nerve in Parkinson’s disease (PD)^49,50^. A more generalized influence of dysbiosis upon the brain has also been proposed in PD^20,51^, Alzheimer’s disease (AD)^21,52,53^ and in multiple sclerosis (MS)^22,54,55^. This has resulted in the testing of dietary modulation of the microbiome or fecal transplants as potential treatments for each of these disorders^20–22,55−57^. Nevertheless, separating cause and effect has proved difficult, and many of these interventions have proven inconclusive. Whether similar events occur in CLN2 disease or other forms of NCL is unclear.

The effects of a dysregulated gut microbiome upon the CNS have been extensively explored^17–22^, and the potential influence of dysbiosis upon the ENS is now also a topic of active investigation^47,48,58–60^. Given that ENS dysfunction has been suggested as a driver of CNS neurodegeneration^61–63^, a local effect of such dysbiosis upon enteric neurons, glia or other bowel cell types may also indirectly influence events within the CNS. Previously, we have characterized degeneration in the brains of *Tpp1*^*R207X/R207X*^ mice and have used CNS-directed therapies to treat this^29^. More recently we also showed neurodegeneration in the ENS of *Tpp1*^*R207X/R207X*^ mice results in bowel dysmotility^14^ but is treatable by intravenous gene therapy^14^. This treatment of the bowel extends lifespan, suggesting that enteric nervous system degeneration in *Tpp1*^*R207X/R207X*^ mice^14^ is life-threatening. Nevertheless, whether the gut-brain axis is altered in CLN2 disease or if the dysregulated microbiome of *Tpp1*^*R207X/R207X*^ mice^15,16^ contributes to disease progression is yet to be resolved. In this study we investigated the potential influence of the previously defined dysregulation of the gut microbiome in *Tpp1*^*R207X/R207X*^ mice^15,16^ upon bowel and brain pathology. This could plausibly be done in several different ways, and before considering housing *Tpp1*^*R207X/R207X*^ mice in a germ-free environment or performing fecal transplants, we first sought to obtain proof-of-concept evidence for whether disrupting the gut microbiota had any influence upon CLN2 disease-associated CNS or ENS phenotypes. As a simple and commonly used approach to explore the influence of the gut microbiome^23–27^, we treated our mice with the VNAM antibiotic cocktail. This approach is not without its limitations, and our study was not sufficiently powered to study differences in gut microbe populations statistically, something which will require much larger and costly experiments to properly define the effects of TPP1 deficiency upon the gut microbiome. Nevertheless, our data reveal that even transient one week-long administration of this combination of four antibiotics markedly altered the composition of the gut microbiota in both WT and *Tpp1*^*R207X/R207X*^ mice. These effects of VNAM are evident acutely after exposure and although recolonization of the gut microbiome does occur to some extent, the effects of VNAM administration persisted for several months. Further analyses utilizing higher numbers of mice will be required to properly confirm the extent and durability of these changes. It is also worth noting that we originally planned VNAM treatment over a much longer period, but this caused sudden early fatality of several treated *Tpp1*^*R207X/R207X*^ mice in a pilot study. This may be associated with the inhibition of GABAergic neurotransmission that is caused by several individual antibiotic components of the VNAM cocktail^64,65^. This reduced GABA signaling may in turn exacerbate the fatal seizure phenotype of *Tpp1*^*R207X/R207X*^ mice that we recently documented^29^. Nevertheless, the current study allows us to determine whether this manipulation of the gut microbiome had a long-term influence upon CLN2-disease associated pathology in both brain and bowel of *Tpp1*^*R207X/R207X*^ mice.

First considering the impact of VNAM treatment upon the brain, unlike previous studies in models of traumatic brain injury^23–27^ where these antibiotics either increased or had no effect upon the innate immune response to injury, VNAM treatment reduced brain CD68 + microglial immunoreactivity in *Tpp1*^*R207X/R207X*^ mice. This reduction in CD68 staining in VNAM treated *Tpp1*^*R207X/R207X*^ mice was by 78% or 64% in S1BF and VPM/VPL, respectively, brain regions that are severely affected in these mice^29^, but was not accompanied by a similar impact on the disease-associated elevation of the astrocyte marker GFAP in the same brain regions. Such reduction in CD68 + microglia might potentially be due to decreasing the systemic inflammation associated with a ‘leaky gut’ that may accompany a dysregulated gut microbiome^17–19^, although it has not been demonstrated whether such events occur to any appreciable extent in this CLN2 disease mouse model. Nevertheless, it is unlikely that VNAM treatment can be considered to provide any therapeutic benefit in this CLN2 model. Indeed, our data suggest relatively little direct influence of altering the gut microbiome on CNS pathology in *Tpp1*^*R207X/R207X*^ mice.

Within the bowel, our data reveal that only one week of exposure to VNAM immediately post-weaning had a severe and long-lasting effect upon the density of enteric neurons in wildtype mice, reducing their number by approximately half. Such effects are consistent with previous reports of the negative impact of perinatal antibiotics upon the ENS^58,59,65–69^, and have obvious implications for the use of these drugs in children. Our data reveal the deleterious impact of even transient exposure to VNAM during the early post-weaning period also extends to epithelial cells of the villi and crypts and bowel smooth muscle. The profound and long-lasting morphological changes we observed in wildtype mice following VNAM treatment highlight the significant impact of antibiotic exposure on intestinal architecture. Previous studies have demonstrated that early-life antibiotic exposure fundamentally alters intestinal epithelial cell composition, functioning, and maturation^66–72^, which may serve as the basis for health problems later in life. The structural intestinal abnormalities we observed are consistent with reports showing that prolonged antibiotic use can cause lasting damage to the intestinal epithelium^71,72^. Importantly, these morphological changes in VNAM-treated wildtype mice closely resembled those seen in untreated *Tpp1*^*R207X/R207X*^ mice, underscoring the severity of antibiotic-induced intestinal damage and raising important concerns about the long-term consequences of early antibiotic exposure in clinical settings^71,72^. The fact that such structural alterations can persist for months after cessation of antibiotic treatment emphasizes the need for judicious use of broad-spectrum antibiotics, particularly during critical developmental periods^68–74^.

Given to the profound effect of VNAM upon the bowel of wildtype mice, it might be anticipated that the same treatment would worsen the already compromised bowel phenotypes we have documented in *Tpp1*^*R207X/R207X*^ mice^14^. At the early post-weaning time when VNAM was administered, the ENS of *Tpp1*^*R207X/R207X*^ mice appears morphologically normal^14^, and subsequently enteric neurons degenerate progressively culminating in approximately 50% of enteric neurons being lost at disease endstage at 14 weeks of age^14^. Remarkably, VNAM treatment did not exacerbate this enteric neuron loss in *Tpp1*^*R207X/R207X*^ mice and even had a small but significant effect protective upon myenteric plexus enteric neurons in the ileum of these VNAM treated *Tpp1*^*R207X/R207X*^ mice. The basis of this effect and why it is not evident in other bowel regions of these mice both warrant further investigation. Nevertheless, administration of VNAM did not worsen the impact of TPP1 deficiency upon villi, crypts and bowel smooth muscle, which we have documented for the first time in this study. These data suggest that neither the gut microbiota, nor its dysregulation that is evident in these *Tpp1*^*R207X/R207X*^ mice^15,16^, drives their bowel phenotypes. Indeed, it is plausible that changes in the gut microbiome may be a consequence of these enteric phenotypes, rather than their cause. Proving this mechanistically is beyond the scope of the current study, which was intended to be a prelude to more detailed mechanistic studies to establish the role of the gut microbiome in CLN2 pathogenesis, both within the bowel and brain. However, our proof-of-concept transient antibiotic ablation studies suggest there is relatively little influence of modulating the gut microbiome upon bowel or brain phenotypes in *Tpp1*^*R207X/R207X*^ mice. However, among the many data points collected there were isolated examples of VNAM treatment producing moderate, but significant beneficial effects in *Tpp1*^*R207X/R207X*^ mice upon enteric neuron density in the ileum and upon microglial activation in vulnerable brain regions. It will be important to explore the basis of these effects mechanistically to determine how they are mediated. Collectively, our data suggest that the marked alterations in the gut microbiome evident in these mice may occur secondary to their profound enteric disease. Given that this enteric neurodegeneration in CLN2 disease is treatable by gene therapy^14^ suggests it is the direct result of TPP1 deficiency within the ENS, and we plan to investigate whether this gene therapy approach also corrects the altered gut microbiome. The effects of TPP1 deficiency appear to be intrinsic to the bowel^14^, but it cannot be excluded that an indirect effect of CNS neurodegeneration upon the bowel may also play a role. This issue is complex to dissect mechanistically, as *Tpp1*^*R207X/R207X*^ mice still die from fatal spontaneous seizures if their CNS is not treated^29^, even if their bowel is effectively treated by gene therapy^14^. We are currently exploring the cellular autonomy of TPP1 deficiency^75^ in both the bowel and brain, but it appears that enteric neurons are more impacted than enteric glia^14^, which also were not influenced by VNAM treatment.

In summary, our findings provide evidence that, despite the profound influence of the gut microbiota on immune modulation and neurodegeneration in other disorders^17–22,61−63^, short-term disruption of the microbiome via VNAM antibiotic administration does not exacerbate either ENS or CNS disease progression in *Tpp1*^*R207X/R207X*^ mice. While VNAM-induced modulation of the gut microbiome resulted in altered microbial diversity and produced significant histological alterations in the ENS of wildtype mice, no significant exacerbation of intestinal morphology, enteric neuron survival, or central nervous system pathology was evident in VNAM-treated  *Tpp1*^*R207X/R207X*^ mice. Indeed, our data reveal isolated examples of restricted benefit of modulating the gut microbiome upon selected features of both bowel and brain pathology that will need to be defined in larger more properly powered studies to determine how these effects are mediated and if they provide any therapeutic benefit. Collectively, our observations suggest that the primary driver of gastrointestinal and neurological dysfunction in CLN2 disease is most likely the underlying TPP1 mutation, rather than being driven primarily by alterations in the gut microbiota. Interestingly, the observed decrease in microglial activation in VNAM treated *Tpp1*^*R207X/R207X*^ mice raises the possibility that antibiotic-induced changes in systemic immunity or metabolism may influence neuroinflammatory pathways. This highlights the complexity of gut-brain interactions and underscores the need for further mechanistic studies to unravel the multifaceted contributions of microbiota-host interactions in the pathogenesis of lysosomal storage disorders.

## Supplementary Information

Below is the link to the electronic supplementary material.

Supplementary Material 1

Supplementary Material 2

Supplementary Material 3

## Data Availability

The raw data used and/or analyzed during the current study are contained in the supporting data file. The 16 S rRNA raw sequencing datasets generated and/or analyzed during the current study are available in the NCBI Sequence Read Archive (SRA) repository, under BioProject accession number PRJNA1369496. Individual sample IDs for these data are listed in Supplementary Data Table 1.
