# Supplementary material for: Antibiotic treatment reveals the contributions of the gut microbiome to CLN2 disease in the central and enteric nervous system
Source: Sci Rep. 2026 Apr 25;16:19161. doi: 10.1038/s41598-026-49850-z (PMC13279791; doi:10.1038/s41598-026-49850-z)
Supplement: Supplementary file 1 — Supplementary Material 1 [file 41598_2026_49850_MOESM1_ESM.docx]

**Table S1:** List of antibodies used.

| **Antibody** | **Concentration** | **Catalog number** | **Source** |
| --- | --- | --- | --- |
| ANNA-1 (HuC/D) | 1:10000 (mouse bowel) | N/A | Kind gift from Dr. Vanda Lenon, Mayo Clinic; RRID: AB_2314657 |
| Mouse anti HuC/D | 1:200 (human bowel) | A21271 | Invitrogen  RRID: AB_221448 |
| Rabbit anti-GFAP | 1:1000 (mouse brain) | Z0334 | Agilent  RRID: AB_10013382 |
| Rat anti-CD68 | 1:400 (mouse brain) | MCA1957 | Biorad  RRID: AB_322219 |
| Rabbit anti-S100 beta | 1:300 (mouse bowel) | ab52642 | Abcam  RRID: AB_882426 |
| Rabbit anti-SCMAS | 1:400 (mouse brain) | ab181243 | Abcam  RRID: AB_2935765 |
| AlexaFluor goat anti-human 546 | 1:400 (mouse bowel) | A-21089 | ThermoFisher Scientific (Invitrogen)  RRID: AB_2535745 |
| AlexaFluor goat anti-rat 546 | 1:400 (mouse brain) | A-11081 | ThermoFisher Scientific (Invitrogen)  RRID: AB_2534125 |
| AlexaFluor goat anti-rabbit 488 | 1:400 (mouse bowel & brain) | A-11008 | ThermoFisher Scientific (Invitrogen)  RRID: AB_143165 |
